# Joint effects of prenatal exposure to indoor air pollution and psychosocial factors on early life inflammation

**DOI:** 10.1016/j.envres.2024.118822

**Published:** 2024-07-01

**Authors:** Grace M. Christensen, Michele Marcus, Petrus J.W. Naudé, Aneesa Vanker, Stephanie M. Eick, W. Michael Caudle, Susan Malcolm-Smith, Shakira F. Suglia, Howard H. Chang, Heather J. Zar, Dan J. Stein, Anke Hüls

**Affiliations:** a Department of Epidemiology, Rollins School of Public Health, Emory University, Atlanta, GA, USA; b Gangarosa Department of Environmental Health, Rollins School of Public Health, Emory University, Atlanta, GA, USA; c Department of Psychiatry and Mental Health, University of Cape Town, Cape Town, South Africa; d Neuroscience Institute, University of Cape Town, Cape Town, South Africa; e Department of Paediatrics and Child Health, Red Cross War Memorial Children's Hospital, University of Cape Town, Cape Town, South Africa; f Department of Biostatistics and Bioinformatics, Rollins School of Public Health, Emory University, Atlanta, GA, USA; g South African Medical Research Council (SAMRC) Unit on Risk and Resilience in Mental Disorders, University of Cape Town, Cape Town, South Africa

**Keywords:** Air pollution, Psychosocial factors, Inflammatory markers, Neuroinflammation

## Abstract

It is hypothesized that air pollution and stress impact the central nervous system through neuroinflammatory pathways Despite this, the association between prenatal exposure to indoor air pollution and psychosocial factors on inflammatory markers in infancy has been underexplored in epidemiology studies. This study investigates the individual and joint effects of prenatal exposure to indoor air pollution and psychosocial factors on early life inflammation (interleukin-1β (IL-1β), interleukin-6 (IL-6), and tumor necrosis factor-α (TNF-α)). We analyzed data from the South African Drakenstein Child Health Study (N = 225). Indoor air pollution and psychosocial factor measurements were taken in the 2nd trimester of pregnancy. Circulating inflammatory markers (IL-1β, Il-6, and TNF-α) were measured in serum in the infants at 6 weeks postnatal. Linear regression models were used to investigate associations between individual exposures and inflammatory markers. To investigate joint effects of environmental and psychosocial factors, Self-Organizing Maps (SOM) were used to create exposure profile clusters. These clusters were added to linear regression models to investigate the associations between exposure profiles and inflammatory markers. All models were adjusted for maternal age, maternal HIV status, and ancestry to control for confounding. Most indoor air pollutants were positively associated with inflammatory markers, particularly benzene and TNF-α in single pollutant models. No consistent patterns were found for psychosocial factors in single-exposure linear regression models. In joint effects analyses, the SOM profile with high indoor air pollution, low SES, and high maternal depressive symptoms were associated with higher inflammation. Indoor air pollutants were consistently associated with increased inflammation in both individual and joint effects models, particularly in combination with low SES and maternal depressive symptoms. The trend for individual psychosocial factors was not as clear, with mainly null associations. As we have observed pro- and anti-inflammatory effects, future research should investigate joint effects of these exposures on inflammation and their health effects.

## Highlights

• Prenatal benzene exposure was associated with increased TNF-α in infancy.
• Prenatal psychiatric distress associated with decreased IL-6 and TNF-α in infancy.
• Profile with high air pollution and depression associated with increased inflammation.

**Sources of** support**:** The Drakenstein Child Health Study was funded by the 10.13039/100000865Bill & Melinda Gates Foundation (10.13039/100000087OPP 1,017,641), DiscoveryFoundation, 10.13039/501100001322South African Medical Research Council, 10.13039/501100001321National Research Foundation South Africa, CIDRI Clinical Fellowship and 10.13039/100010269Wellcome Trust (204755/2/16/z). AH and SME were supported by the HERCULES Center (10.13039/100000066NIEHS P30ES019776). AH was also supported by the 10.13039/100000049National Institute on Aging (10.13039/100000049NIA R01AG079170). 10.13039/100014559GMC was supported by the 10.13039/100000066NIEHS T32 Training Program in Environmental Health and Toxicology (5T32ES12870).

**Table 1:** Descriptive characteristics of the study population.

N	225
Maternal Age (mean (SD))	27.65 (5.98)
Male Child (%)	130 (57.8)
Ancestry	
Black African Ancestry (5)	135 (60.0)
Mixed Ancestry (%)	90 (40.0)
Mother HIV-infected (%)	95 (42.2)
CD4 Nadir values among HIV-infected mothers	
<350 cells/mm^3^	18 (39.1)
350–500 cells/mm^3^	7 (15.2)
≥500 cells/mm^3^	21 (45.7)
Indoor Air Pollutants	
PM10 μg/m3 (median [IQR])	39.01 [14.97, 69.62]
CO mg/m3 (median [IQR])	0.00 [0.00, 120.00]
Benzene μg/m3 (median [IQR])	4.34 [1.91, 12.72]
Toluene μg/m3 (median [IQR])	16.02 [6.61, 45.02]
NO2 μg/m3 (median [IQR])	6.05 [3.10, 11.27]
SO2 μg/m3 (median [IQR])	0.00 [0.00, 0.14]
Psychosocial Factors	
Urine Cotinine ng/ml (median [IQR])	52.70 [14.60, 500.00]
SES Asset Sum (median [IQR])	7.00 [5.00, 8.00]
Food Insecurity Total Score (median [IQR])	0.00 [0.00, 3.00]
SRQ-20 Total Score (median [IQR])	4.00 [2.00, 8.00]
Emotional IPV Score (median [IQR])	5.00 [4.00, 7.00]
Physical IPV Score (median [IQR])	6.00 [5.00, 8.00]
LEQ Total Score (median [IQR])	2.00 [1.00, 3.00]
EPDS Total Score (median [IQR])	10.00 [7.00, 14.00]
ASSIST Tobacco Score (median [IQR])	0.00 [0.00, 15.00]
ASSIST Alcohol Score (median [IQR])	0.00 [0.00, 0.00]
Inflammatory Markers	
IL-1β pg/mL (median [IQR])	1.05 [0.48, 1.76]
IL-6 pg/mL (median [IQR])	1.93 [0.69, 4.08]
TNF-α pg/mL (median [IQR])	19.57 [14.48, 28.23]

Abbreviations: Particulate Matter (PM10); Carbon monoxide (CO); Nitrogen dioxide (NO2); Sulfur dioxide (SO2); Socioeconomic Status (SES); Self-Reporting Questionnaire (SRQ-20); Edinburgh Postnatal Depression Scale (EPDS); Life Experiences Questionnaire (LEQ); Intimate Partner Violence (IPV); Alcohol, Smoking, and Substance Involvement Screening Test (ASSIST); Interleukin 1β (IL-1β); Interleukin 6 (IL-6); Tumor Necrosis Factor-α (TNF-α).

## 1 Introduction

During everyday life pregnant individuals are exposed to a variety of environmental and psychosocial stressors, including indoor air pollution, violence and food insecurity. Exposure to air pollution and psychosocial stress during pregnancy have been associated with a variety of adverse fetal, infant and child health outcomes from preterm birth and respiratory illness to altered neurodevelopment (Beijers et al., 2014; Vrijheid et al., 2016; Friedman et al., 2021). It is hypothesized that the maternal and infant inflammatory response contributes to this association.

Animal models have shown that air pollutants cause a systemic inflammatory response (Block and Calderón-Garcidueñas, 2009). Both the physical air pollutant particle, and the toxic components absorbed on the particle can create an inflammatory response. Translocation of air pollutant particles from the lungs and nasal pathways to other areas of the body cause damage to the body and create an inflammatory response (Block and Calderón-Garcidueñas, 2009). Air pollution particles have also been shown to cross the placenta and directly reach the developing fetal body (Johnson et al., 2021). However, there are few studies in humans investigating how prenatal exposure to air pollutants impacts inflammatory response in the infant.

Childhood stress has been associated with chronic peripheral inflammation in both cross-sectional and longitudinal studies (Olvera Alvarez et al., 2018). A meta-analysis found low adulthood SES was associated with higher levels of systemic inflammation in adults, including higher levels of interlukin-6 (IL-6) (Muscatell et al., 2020). However, there is little epidemiologic research on how psychological stress during pregnancy impacts inflammation in the child. The few epidemiology studies available show mixed pro- and anti-inflammatory results (Hahn et al., 2019; Pedersen et al., 2018).

As pregnant individuals are often exposed to both air pollution and stress-inducing psychosocial factors, joint effects are likely and have been seen in prior studies investigating other health effects ((Christensen et al., 2023); Eick et al., 2022; Padula et al., 2020). As of this writing, only one epidemiology study has investigated joint effects of prenatal exposure to air pollution and psychosocial factors on infant inflammation. Hahn et al. investigated effect modification of the association between prenatal fine particulate matter (PM_2.5_) exposure and inflammatory markers in cord blood by maternal depression. They found no significant effect modification of the association (Hahn et al., 2021). More epidemiological studies are needed to investigate both the individual and joint effects of prenatal exposure to air pollution and psychosocial factors on markers of inflammation.

Our study leverages data from a South African birth cohort to investigate how individual and joint prenatal exposure to indoor air pollution and psychosocial factors are associated with inflammatory markers, specifically interleukin-1β, interleukin-6, and tumor necrosis factor-α, in the infant at 6 weeks postnatal. This population from a low-to middle-income country is highly exposed to both indoor air pollution as well as psychosocial stressors, which makes joint effects of these exposure likely (Christensen et al., 2023; Vanker et al., 2015; Stein et al., 2015). We use self-organizing maps to create exposure profiles of prenatal exposure to indoor air pollutants and psychosocial factors. Then we investigate the association between prenatal exposure profile and infant inflammatory markers. This study hypothesizes that prenatal exposure to increased levels of both indoor air pollution and psychosocial factors will increase levels of infant inflammatory markers.

## 2 Methods

### 2.1 Study population

This study is based on a subset of participants from the Drakenstein Child Health Study (DCHS). The DCHS is a multi-disciplinary population-based pregnancy cohort based in South Africa. Recruited during the 2nd trimester of pregnancy, women 18 years and older were enrolled from 2012 to 2015. Follow-up with mother-child pairs was conducted at multiple points in the child's first year of life and annually thereafter. More detailed recruitment and follow up information can be found elsewhere (Stein et al., 2015; Zar et al., 2015). Of the N = 1141 mother-child pairs recruited, a subset of n = 225 were included in this analysis. The reduced sample size in this analysis is due to the small subset of infants selected for measurement of inflammatory markers at 6 weeks old. Infants were selected by convenience sampling. Based on research priorities at the time, the subset of DCHS participants selected for measurement of inflammatory markers was enriched with HIV infected mothers and an equal number of non-infected mothers (Fig. S1, Table S1). Further inclusion criteria for this analysis included having measurements of indoor air pollution and psychosocial factors in the second trimester of pregnancy. Due to infection prevention efforts, only two children were born with HIV. HIV infected children were excluded from this analysis sample (Fig. S1). Mothers infected with HIV were started on antiretroviral therapy upon entering the DCHS. The DCHS was approved by the Human Research Ethics Committee (HREC) of the University of Cape Town (HREC 401/2009), Stellenbosch University (N12/02/0002) and Western Cape Provincial Health Research Committee (2011RP45). Written informed consent was provided by each mother for herself and her child and is renewed annually.

### 2.2 Indoor air pollution assessment

As described previously (Christensen et al., 2023 ; Vanker et al., 2015; Zar et al., 2015), indoor air pollution measurements were taken during participants’ 2nd trimester of pregnancy. Pollutants measured include particulate matter <10 μm in diameter (PM_10_), carbon monoxide (CO), nitrogen dioxide (NO_2_), sulfur dioxide (SO_2_), and Volatile Organic Compounds (VOCs) benzene and toluene. Particulate Matter (PM_10_) was collected over 24 h with a personal air sampling pump (SKC AirChek 52®), using a gravimetrically pre-weighted filter. Carbon monoxide (CO) was collected over 24 h using an Altair® carbon monoxide single gas detection unit, electrochemical sensor detection of gas at 10-min intervals were collected. Sulfur dioxide (SO_2_) and nitrogen dioxide (NO_2_) were collected over 2 weeks using Radiello® absorbent filters in polyethylene diffusive body. Volatile organic compounds, including benzene and toluene, were collected over 2 weeks using Markes® thermal desorption tubes (Vanker et al., 2015). More detailed indoor air pollution exposure assessment methods have been published elsewhere (Vanker et al., 2015). Additional information on factors that could impact indoor air pollution (e.g., type of home, distance from major road, size of home, number of inhabitants, access to basic amenities, fuels used for cooking and heating, ventilation within homes, and pesticides and cleaning materials used in the home) was collected at home visits (Vanker et al., 2015).

### 2.3 Assessment of psychosocial factors

Assessment of psychosocial factors was conducted via self-reported questionnaire in the 2nd trimester of pregnancy. Employment, education, household income, household assets, marital status, number of dependents, and financial activities were included as indicators of socioeconomic status. Perceived household food insecurity was assessed using an adapted version of the USDA Household Food Security Scale (Pellowski et al., 2017). Intimate partner violence (IPV) was assessed using the IPV Questionnaire adapted from the World Health Organization (WHO)'s multi-country study and the Women's Health Study in Zimbabwe (Shamu et al., 2011; Jewkes, 2002). The IPV questionnaire assesses lifetime and recent (past year) exposure to emotional, physical, and sexual violence. The World Mental Health Life Events Questionnaire (LEQ) was used to measure trauma and resilience. Use of alcohol and tobacco were assessed using the Alcohol, Smoking, and Substance Involvement Screening Test (ASSIST). Additionally, tobacco smoke exposure was assessed via urinary cotinine. The Self Reporting Questionnaire (SRQ-20), a measure endorsed by the WHO, was used to measure psychological distress (Beusenberg et al., 1994; van der Westhuizen et al., 2016). The Edinburgh Postnatal Depression Scale (EPDS) was used to measure depressive symptoms (Murray and Cox, 1990).

### 2.4 Assessment of inflammatory markers

Peripheral blood serum samples from the infants at 6 weeks old were collected as previously described (Zar et al., 2015). Pro-inflammatory immune markers (IL-1β, IL-6, and TNF-α) are analyzed with a Milliplex® Luminex premix 13-plex kit (HSTCMAG28SPMX13; Merck) (Zar et al., 2015). IL-1β, IL-6, TNF-α were selected *a priori* as inflammatory markers for these analyses as they are commonly reported in literature on their associations with neuroinflammation (Block and Calderón-Garcidueñas, 2009; Olvera Alvarez et al., 2018). The intra- and inter-assay coefficients of variations for IL-1β, IL-6 and TNF-α were <7% and <14%. The lower limit of detection for IL-1β, IL-6 and TNF-α were 0.14 pg/mL, 0.11 pg/mL and 0.16 pg/mL respectively. Measures that were below the lower limit of detection (IL-1β (n = 6) and IL-6 (n = 10)) were replaced by values representing a half of the lowest value of on the standard curve in an effort to avoid statistical bias (Pfister et al., 2020).

IL-1β, IL-6 and TNF-α had a skewed distribution and were natural log-transformed in all statistical analyses.

### 2.5 Statistical analysis

#### 2.5.1 Multiple imputation of missing values

While there were no missing values in the outcome or covariates, some participants were missing exposure data including certain indoor air pollutants and psychosocial factors (Table S2). Based on inspections of missingness patterns, we assume these values are missing at random (Fig. S2). Using the *hmisc* R package, multiple imputation was preformed to impute missing exposure variables. Exposure variables were imputed using predictive mean matching, models included pre- and postnatal (4 months) measurements of indoor air pollution, psychosocial factors, and household characteristics. Five seed numbers were created using a random number generator, each seed resulted in its own set (k = 10) of variables with missing values imputed. One of the k sets was randomly selected to use for analyses as pooling of the k sets was not compatible with one method used in the statistical analysis. The seed with the highest R^2^ values, a measure available within *hmisc* used to explain how well missing values were predicted, was selected for use in primary analyses and results. Sensitivity analyses with complete cases and other imputation seeds were also conducted.

#### 2.5.2 Association analyses

To investigate the association between prenatal exposure to indoor air pollution and psychosocial factors with inflammation at 6 weeks old, we used both single-exposure and environmental mixture methodology. Traditional single-exposure linear regression modeling techniques were used to estimate the associations of all exposures individually with inflammation. Next, using Self-Organizing Maps (SOM) we estimated joint effects of indoor air pollution and psychosocial factor exposures on inflammation. These joint effects estimated by the SOM can highlight exposure profiles of our population that have the greatest impact on inflammation in the infant.

#### 2.5.3 Single-exposure models

To investigate how individual prenatal indoor air pollutant and psychosocial factors are associated with inflammation at 6 weeks old we used single-exposure linear regression models. All exposures were right skewed and natural log-transformed for linear regression analyses. Each exposure was used in its own linear regression model adjusted for the minimal set of confounders, which included maternal age, maternal HIV status, ancestry, and socioeconomic status (except when the exposure of interest). Confounders were determined using a directed acyclic graph (DAG; Fig. S3). In sensitivity analyses, single-exposure models were additionally adjusted for principal components (PCs) of the other group of exposures (indoor air pollutants or psychosocial factors). We used PCs as confounding variables, created by principal components analysis (PCA), as opposed to the individual variables to include information on many correlated variables but avoid over adjustment of the model. In these models the first 4 PCs of indoor air pollutants or psychosocial stressors, respectively, were included in the models as confounders. More details on the use of PCA in controlling for confounding can be found in the supplementary materials, including scree plots (Fig. S4).

We explored effect modification by ancestry (Black African vs Mixed Ancestry), and maternal HIV status. Ancestry was chosen as an effect modifier because there are noted differences in socioeconomic as well as psychosocial risk factors by ancestry in the DCHS (Stein et al., 2015). HIV status was also investigated as an effect modifier because of the known connection with inflammation (Sevenoaks et al., 2021) and the high burden in this population.

#### 2.5.4 Self-organizing maps

Self-organizing maps (SOM) is an unsupervised algorithm that creates profiles of exposure. SOM was used to create profiles of prenatal exposure to indoor air pollution and psychosocial factors. SOM identifies clusters of exposure, or profiles of exposure, that are homogeneous within cluster and heterogeneous between clusters (Pearce et al., 2016, 2021). To prepare for the SOM algorithm, exposures were natural log-transformed and scaled to have a mean of 0 and a standard deviation of 1. The number of clusters chosen for association analyses was based on statistical measures of group structure, including Akaike information criterion (AIC), and adjusted R^2^. As has been done previously, visual inspection of the clusters for interpretability and appropriate distribution of participants among clusters was also used to select the number of clusters. To investigate the association between the SOM clusters and inflammation, SOM clusters were assigned to participants and added to linear regression models as a categorical exposure variable. The linear regression model using SOM clusters as the exposure was adjusted for maternal age, maternal HIV status, and ancestry. We used the SOM R package as implemented in https://github.com/johnlpearce/ECM.

All analyses were performed using R version 4.1.2 (R Core Team, Vienna, Austria).

## 3 Results

On average, mothers in our study population of n = 225 mother-child pairs were 27.7 years old (SD: 5.98). Over 40% of mothers were infected with HIV, and had high CD4 nadir values prior to starting antiretroviral therapy. About half (57.8%) of the children included in this analysis were male (Table 1). Indoor air pollutants were not highly correlated, while psychosocial factors were moderately correlated with each other. Indoor air pollutants and psychosocial factors were not correlated with each other (Table S3). There was low to moderate correlation (ρ = 0.05–0.39) among inflammatory markers (Table S4).

**Fig. 1:**
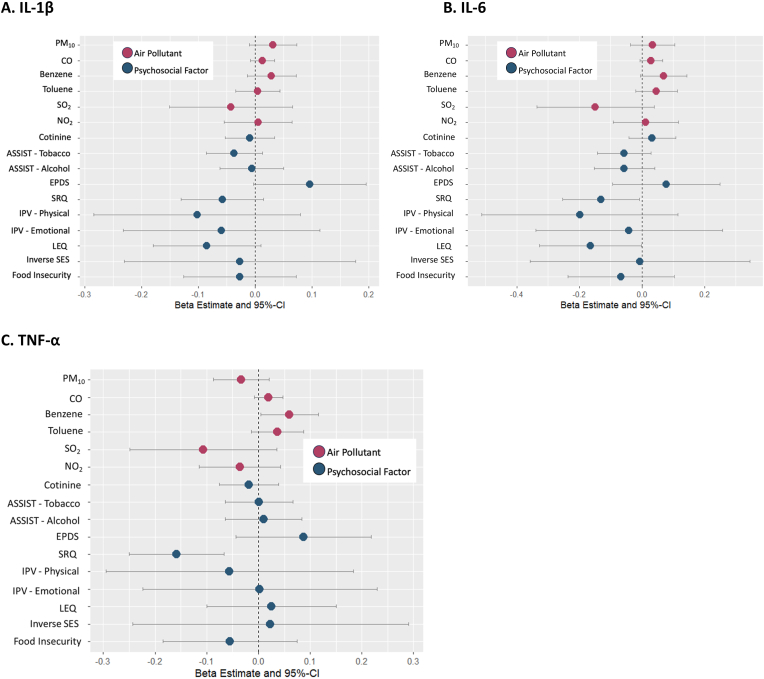
Single-exposure linear regression models of the association between prenatal indoor air pollution and psychosocial factor exposures on inflammatory markers at 6 weeks postnatal. All linear regression models were adjusted for maternal age, maternal HIV status, ancestry and socioeconomic status (when not the exposure of interest). **A**. Models using Il-1β as the outcome. **B.** Models using Il-6 as the outcome. **C.** Models using TNF-α as the outcome. Abbreviations: Particulate Matter (PM10); Carbon monoxide (CO); Nitrogen dioxide (NO2); Sulfur dioxide (SO2); Socioeconomic Status (SES); Self-Reporting Questionnaire (SRQ-20); Edinburgh Postnatal Depression Scale (EPDS); Life Experiences Questionnaire (LEQ); Intimate Partner Violence (IPV); Alcohol, Smoking, and Substance Involvement Screening Test (ASSIST); Interleukin 1β (IL-1β); Interleukin 6 (IL-6); Tumor Necrosis Factor-α (TNF-α).

In single-exposure linear regression models adjusted for confounders, there were consistent positive associations between most indoor air pollutants and inflammatory markers. For example, a one-unit increase in benzene was associated with higher TNF-α levels ([beta: 0.06; 95% CI: 0.00, 0.12]; Table S4, Fig. 1C). SO_2_ was the only air pollutant which was associated with lower inflammation, though these associations were not statistically significant (Table S5, Fig. 1).

No consistent patterns were found for psychosocial factors in single-exposure linear regression models. A majority of psychosocial factors were not associated with any inflammatory marker. Psychological distress (SRQ) was consistently associated with lower inflammation (IL-6: beta: −0.13; 95% CI: −0.25, −0.01; TNF-α: beta: −0.16; 95% CI: −0.25, −0.07). A one unit increase in adverse life experiences (LEQ) (beta: −0.16; 95% CI: −0.33, −0.00) was also associated with lower IL-6 levels (Table S5, Fig. 1). Depression symptoms (EPDS) were consistently associated with higher inflammation, though none of these effect estimates were statistically significant. Results were similar in sensitivity analyses when additionally adjusting for PCs of the other exposure group (Table S6, and in sensitivity analyses using complete cases and when using other MI seeds in the multiple imputation (Table S5).

In effect modification analyses, the effect of psychological distress (SRQ) on inflammatory markers was stronger in Black African participants compared to mixed ancestry participants (Figs. S5A and S5D; effect modification statistically significant for IL-6). There was no significant effect modification by HIV status (Fig. S6).

**Fig. 2:**
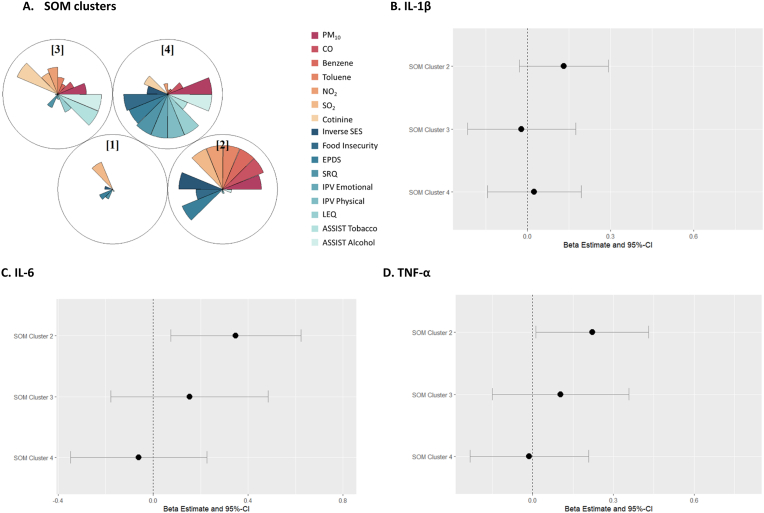
Results from self-organizing map (SOM) analysis using prenatal indoor air pollutants and psychosocial factors. **A.** SOM clusters created using pre-natal indoor air pollutants and psychosocial factors. **B.** Associations between SOM clusters and IL-1β, adjusted by maternal age, maternal HIV status, and ancestry. SOM cluster 1 is used as the reference group. **C.** Associations between SOM clusters and IL-6, adjusted by maternal age, maternal HIV status, and ancestry. SOM cluster 1 is used as the reference group. **D.** Associations between SOM clusters and TNF-α, adjusted by maternal age, maternal HIV status, and ancestry. SOM cluster 1 is used as the reference group. Abbreviations: Particulate Matter (PM10); Carbon monoxide (CO); Nitrogen dioxide (NO2); Sulfur dioxide (SO2); Socioeconomic Status (SES); Self-Reporting Questionnaire (SRQ-20); Edinburgh Postnatal Depression Scale (EPDS); Life Experiences Questionnaire (LEQ); Intimate Partner Violence (IPV); Alcohol, Smoking, and Substance Involvement Screening Test (ASSIST); Interleukin 1β (IL-1β); Interleukin 6 (IL-6); Tumor Necrosis Factor-α (TNF-α).

SOM analysis grouped our study population into 4 exposure profiles, which characterize different distributions of the joint exposure to environmental and psychosocial factors. Cluster 1 was selected as the reference cluster in regression analyses because it had the lowest exposure to all psychosocial factors and indoor air pollutants, except for SO_2_ (Table S7, Fig. 2A). Compared to cluster 1, cluster 2, a profile with high indoor air pollution, low SES, and high depression symptoms (EPDS) was associated with higher inflammation. Associations were strongest for IL-6 (beta: 0.35; 95% CI: 0.07, 0.62) and TNF-α (Beta: 0.22; 95% CI: 0.01, 0.43) and smaller for IL-1β (beta: 0.13; 95% CI: −0.03, 0.29) (Table S8, Fig. 2). Cluster 3 (high cotinine and ASSIST tobacco and alcohol scores) and cluster 4 (high levels of psychosocial factors and PM_10_) were not associated with any inflammatory marker, compared to cluster 1 (Table S8, Fig. 2).

## 4 Discussion

In our study of prenatal exposure to indoor air pollutants and psychosocial factors on markers of neuroinflammation at 6 weeks old in the South African DCHS, indoor air pollutants were consistently associated with increased inflammatory markers, IL-1β, IL-6 and TNF-α. This positive association was seen across the three inflammatory markers measures, and in both individual and joint effects models. Specifically, benzene was positively associated with increased TNF-α in single-exposure models, and the SOM cluster associated with high indoor air pollution and depression symptoms was associated with IL-6 and TNF-α. The trend for psychosocial factors was less clear.

Both individual and joint effects models identified a positive trend between prenatal indoor air pollution and all inflammatory markers. This trend has also been seen in some other studies investigating prenatal exposure to air pollution and inflammation, however findings in the literatures are mixed. There is evidence of prenatal exposure to air pollution impacting inflammatory markers in cord blood, a proxy for infant inflammation. One study investigating traffic-related air pollution exposure during pregnancy in a Spanish cohort found NO_2_ and PM_10_ were associated with increased odds of detecting IL-1β and IL-6 in cord blood (García-Serna et al., 2022). Another study found PM_10_ exposure during the last 3 months of pregnancy was associated with increased IL-1β levels in cord blood (Latzin et al., 2011). Another study, investigating prenatal exposure to traffic-related air pollutants found PM_2.5_ was associated with decreased IL-6, TNF-α, and IL-10 levels in cord blood (Hahn et al., 2021). A majority of the association between air pollutants and inflammation in pregnant individuals only measure inflammation in cord blood samples, rather than in infants, as was done in our study. Another study found comparing pre- and postnatal exposure to air pollution and inflammation found only postnatal exposure was associated with increased Il-4, IL-5, IL-6, and TNF-α, at 1–2 years old (Deng et al., 2022). As previously mentioned, few studies have investigated inflammation at the same time period as our study. This may be due to difficulty in obtaining enough blood for measurement in infants.

We found prenatal benzene exposure was significantly associated with increased TNF-α. In our study, only benzene had a median value above indoor air standards (Vanker et al., 2015; WHO guidelines for indoor air). To date, no previous study has investigated prenatal exposure to benzene and inflammation. A study investigating human cell responses to benzene found benzene stimulated the production of cytokines in human peripheral blood mononuclear cells (Gillis et al., 2007). However, another study found that adults occupationally exposed to a benzene-toluene-xylene mixture had decreased production of TNF-α (Haro-García et al., 2012). More research needs to be done to determine the association between benzene and inflammation.

The participants in the DCHS have high prevalence of psychosocial stress and depression, as well as high exposure to traumatic stressors and intimate partner violence (Stein et al., 2015). While many of the psychosocial factors investigated in our study were not associated with inflammation at 6-weeks old, prenatal depression had a suggestive positive association with IL-1β, and TNF-α. Few epidemiology studies have investigated prenatal depression in association with inflammation in the child, however, research has shown depressed pregnant women have elevated serum inflammatory cytokines (Azar and Mercer, 2013; Cassidy-Bushrow et al., 2012). Another study in the DCHS found prenatal depression, using the beck Depression Inventory as opposed to the EPDS, was associated with increased inflammatory cytokine levels at 6–10 weeks old (Naudé et al., 2022). Specifically, increased IL-1β levels were associated with prenatal depression, which is in line with our study (Naudé et al., 2022).

Surprisingly, the SRQ-20, a measure of psychological distress, was associated with decreased IL-6, and TNF-α levels at 6 weeks, particularly among mothers of Black African ancestry Adverse life events were also associated with decreased IL-6 levels in our study, particularly among Black African Mothers. In the DCHS, HIV-infected mothers were primarily of Black African ancestry (Stein et al., 2015). In this cohort, maternal HIV infection was associated with lower cytokine levels in the infant at 6 weeks (Sevenoaks et al., 2021). Though in this study we did not see effect modification by HIV status. There were also significant differences in age at enrollment, employment, and partner support between Black African and mixed ancestry mothers in the DCHS (Stein et al., 2015). These differences could be driving the effect modification by ancestry that we found for the associations between psychological distress, adverse life events, and inflammatory cytokines. Another surprising aspect of these findings is that as discussed above, depression was associated with increased inflammation while psychological distress was associated with decreased inflammation. As seen in the SOM analysis, high psychological distress co-occurred with low air pollution exposure in this population. The protective effect of SRQ score may therefore be due to the low exposure to indoor air pollution.

In our joint effects SOM analysis, we found a prenatal exposure profile with high indoor air pollution, low SES, and depression exposure was associated with increased IL-6 and TNF-α. Indicating that indoor air pollution and psychosocial stressors may jointly increase inflammation. This is consistent with a prior study which investigated effect modification of traffic-related air pollutants and inflammation by depression, finding that infants of ever depressed mothers had lower cord blood cytokine concentrations compared to those with never depressed mothers (Hahn et al., 2021). Further work is needed to investigate the precise biological mechanisms underlying how indoor air pollution and psychosocial stressors increase inflammation.

There are several limitations of this study. First, inflammatory measures were only measured in a small subset of the DCHS population. Due to the small sample size, there were wide confidence intervals for many of the individual psychosocial factor associations. A larger sample size would increase power to detect associations among psychosocial factors. There may also be limitations surrounding selection bias with this subsample of the DCHS. The subsample of DCHS mother-child pairs selected for measurement of inflammatory markers was enriched with HIV-infected mothers. Second, exposures were only measured once during the prenatal period and represent the exposure during that entire period. Collecting indoor air pollution measurements is more cost and labor intensive compared to collecting outdoor pollution measurements, and it was not possible to collect these measurements at more than one period during pregnancy. Therefore, indoor air pollution was only measured once towards the end of the second trimester. This may lead to exposure misclassification particularly for the indoor air pollutants. Few studies have investigated sensitive periods of exposure to air pollution during pregnancy and inflammation, but one such study found the first and third trimesters to be windows of higher susceptibility (García-Serna et al., 2022). Future research should investigate sensitive periods of exposure during pregnancy to further explain how pollutants impact the inflammatory response.

An additional limitation is the lack of fine (PM_2.5_) and ultrafine PM measurements which have the largest direct effect on neuroinflammation. Fine and ultrafine PM were not collected during pregnancy for this cohort because at the time it was not feasible for a cohort of this size. PM smaller than PM_10_ are hypothesized to increase systemic and neuroinflammation because their smaller size allows for particles to travel throughout the body and brain (Block and Calderón-Garcidueñas, 2009). Human and animal research has shown microglia, the resident immune cells of the brain, respond to the invading particle damage by releasing inflammatory cytokines such as TNF-α, IL-1β, and IL-6, and reactive oxygen species (ROS) (Block and Calderón-Garcidueñas, 2009). Chronic activation of the microglia and over production of inflammatory markers and ROS can cause neuronal damaging effects (Block and Calderón-Garcidueñas, 2009). Impaired neuronal cell functioning may contribute to CNS disease pathology and development of clinical neurological disorders (Block and Calderón-Garcidueñas, 2009). Inflammatory response from the microglia during gestation and early life periods can also affect brain development through microglial involvement in pruning and shaping of the neuronal synapses (Bollinger and Wohleb, 2019; Paolicelli and Ferretti, 2017). The impact of PM_2.5_ on measures of inflammation in humans is mixed. One study found exposure to ambient PM_2.5_ during pregnancy was associated with increased maternal IL-6 and TNF-α levels, but did was not associated with measures of inflammation on cord blood (Friedman et al., 2021). Another study found PM_2.5_ exposure was not associated with IL-6 and TNF-α levels during pregnancy (Gogna et al., 2021). An *in vitro* study found that in addition to PM size, chemical composition of PM may contribute to the mixed results of epidemiology studies investigating PM exposure and inflammation (Manzano-León et al., 2016). Further research is needed to investigate the impact of PM on inflammation.

This study also has several strengths that are worth highlighting. First, there are few epidemiology studies that investigate prenatal exposure to environmental and psychosocial factors on inflammation in the infant. This study adds valuable insight into the individual and joint effects of prenatal exposure to indoor air pollutants and psychosocial factors on inflammation in the infant. Infancy is an important time period of brain development and shaping of synapses, ad increased inflammation may contribute to CNS disease (Block and Calderón-Garcidueñas, 2009; Bollinger and Wohleb, 2019). Additionally, the DCHS is a unique cohort that provided prospective data from an understudied population. The DCHS also measures a variety of indoor air pollutants and psychosocial factor exposures during pregnancy which allows for estimation of joint effects of these exposures.

## 5 Conclusions

This study identified indoor air pollution exposure during pregnancy as a possible source of increased inflammation in infancy. Reducing maternal exposure to indoor air pollution during pregnancy may reduce levels of infant inflammatory markers, which could consequently reduce incidence of psychopathology in childhood. The association between psychosocial factors and inflammation remains unclear and should be studied in a larger population. Future studies should additionally investigate fine and ultrafine PM exposure. Additionally, future work is needed on the mechanisms underpinning how inflammation may mediate the association between prenatal environmental and psychosocial exposures and outcomes like child neurodevelopment and psychopathology.

## CRediT authorship contribution statement

**Grace M. Christensen:** Writing – review & editing, Writing – original draft, Visualization, Methodology, Investigation, Formal analysis, Conceptualization. **Michele Marcus:** Writing – review & editing, Supervision. **Petrus J.W. Naudé:** Writing – review & editing, Funding acquisition, Data curation. **Aneesa Vanker:** Writing – review & editing, Data curation. **Stephanie M. Eick:** Writing – review & editing. **W. Michael Caudle:** Writing – review & editing. **Susan Malcolm-Smith:** Writing – review & editing, Data curation. **Shakira F. Suglia:** Writing – review & editing. **Howard H. Chang:** Writing – review & editing. **Heather J. Zar:** Writing – review & editing, Funding acquisition, Data curation. **Dan J. Stein:** Writing – review & editing, Funding acquisition, Data curation. **Anke Hüls:** Writing – review & editing, Writing – original draft, Supervision, Methodology, Investigation, Conceptualization.

## Declaration of competing interest

The authors declare the following financial interests/personal relationships which may be considered as potential competing interests: Dan Stein reports a relationship with Discovery Vitality that includes: consulting or advisory. Dan Stein reports a relationship with Johnson & Johnson that includes: consulting or advisory. Dan Stein reports a relationship with Kanna that includes: consulting or advisory. Dan Stein reports a relationship with L'Oreal that includes: consulting or advisory. Dan Stein reports a relationship with Orion that includes: consulting or advisory. Dan Stein reports a relationship with Sanofi that includes: consulting or advisory. Dan Stein reports a relationship with Servier that includes: consulting or advisory. Dan Stein reports a relationship with Takeda that includes: consulting or advisory. Dan Stein reports a relationship with Vistagen that includes: consulting or advisory. The other authors declare that they have no known competing financial interests or personal relationships that could have appeared to influence the work reported in this paper.

## Data Availability

Data will be made available on request.
