# Supplementary material for: Joint effects of prenatal exposure to indoor air pollution and psychosocial factors on early life inflammation
Source: Environ Res. 2024 Jul 1;252:118822. doi: 10.1016/j.envres.2024.118822 (PMC11188991; doi:10.1016/j.envres.2024.118822)
Supplement: Multimedia component 1 [file mmc1.docx]

**Supplementary Materials**

**Joint Effects of Prenatal Exposure to Indoor Air Pollution and Psychosocial Factors on Early Life Inflammation**

Grace M. Christensen^1^, Michele Marcus^1,2^, Petrus J.W. Naudé^3,4^, Aneesa Vanker^5^, Stephanie M. Eick^1,2^, Michael Caudle^2^, Susan Malcolm-Smith^3,4^, Shakira F. Suglia^1^, Howard H. Chang^2,6^, Heather J. Zar^5,7^, Dan J. Stein^3,4,7^, Anke Hüls^1,2^

^1^ Department of Epidemiology, Rollins School of Public Health, Emory University, Atlanta, GA, USA

^2^ Gangarosa Department of Environmental Health, Rollins School of Public Health, Emory University, Atlanta, GA, USA

^3^Department of Psychiatry and Mental Health, University of Cape Town, Cape Town, South Africa

^4^ Neuroscience Institute, University of Cape Town, Cape Town, South Africa

^5^ Department of Paediatrics and Child Health, Red Cross War Memorial Children’s Hospital, University of Cape Town, Cape Town, South Africa

^6^ Department of Biostatistics, Rollins School of Public Health, Emory University, Atlanta, GA, USA

^7^ South African Medical Research Council (SAMRC) Unit on Risk and Resilience in Mental Disorders, University of Cape Town, Cape Town, South Africa

Supplementary Methods

Principal components analysis (PCA) was used to control for confounding in individual exposure models. Specifically, confounding from psychosocial factor exposure variables in models estimating the individual effects of air pollutants and vice versa. We selected 4 principal components to be included as confounders as this accounted for greater than 70% of the variance (Figure S4).

**Tables**

**Table S1**. Comparison of demographic and exposure characteristics between the full DCHS cohort, the subsample with indoor air pollution measurements, and the analysis sample with inflammatory marker measurements.

|  | Full DCHS Cohort | IAP Subsample | Analysis Sample |
| --- | --- | --- | --- |
|  |  |  |  |
| N | 1143 | 819 | 225 |
| Maternal Age (mean (SD)) | 26.60 (5.68) | 26.60 (5.67) | 27.65 (5.98) |
| Male Child (%) | 586 (51.3) | 422 (51.5) | 130 (57.8) |
| Mixed Ancestry (%) | 510 (44.7) | 379 (46.3) | 90 (40.0) |
| Mother HIV Positive (%) | 248 (21.7) | 171 (20.9) | 95 (42.2) |
| Indoor air pollutants |  |  |  |
| PM10 µg/m3 (median [IQR]) | 33.37 [12.49, 64.80] | 33.45 [12.49, 65.43] | 39.01 [14.97, 69.62] |
| CO mg/m3 (median [IQR]) | 0.00 [0.00, 102.50] | 0.00 [0.00, 120.00] | 0.00 [0.00, 120.00] |
| Benzene µg/m3 (median [IQR]) | 4.28 [1.75, 11.29] | 4.34 [1.75, 11.50] | 4.34 [1.91, 12.72] |
| Toluene µg/m3 (median [IQR]) | 16.79 [7.04, 44.24] | 16.94 [7.09, 44.79] | 16.02 [6.61, 45.02] |
| NO2 µg/m3 (median [IQR]) | 7.13 [3.33, 12.69] | 7.19 [3.34, 12.70] | 6.05 [3.10, 11.27] |
| SO2 µg/m3 (median [IQR]) | 0.00 [0.00, 0.28] | 0.00 [0.00, 0.28] | 0.00 [0.00, 0.14] |
| Psychosocial Factors |  |  |  |
| Urine Cotinine ng/ml (median [IQR]) | 43.00 [10.70, 500.00] | 43.35 [10.70, 500.00] | 52.70 [14.60, 500.00] |
| SES Asset Sum (median [IQR]) | 7.00 [5.00, 8.00] | 7.00 [5.00, 8.00] | 7.00 [5.00, 8.00] |
| Food Insecurity Total Score (median [IQR]) | 0.00 [0.00, 2.00] | 0.00 [0.00, 2.00] | 0.00 [0.00, 3.00] |
| SRQ-20 Total Score (median [IQR]) | 4.00 [1.00, 7.00] | 4.00 [1.50, 7.00] | 4.00 [2.00, 8.00] |
| EPDS Total Score (median [IQR]) | 9.00 [6.00, 12.00] | 9.00 [6.00, 13.00] | 10.00 [7.00, 14.00] |
| LEQ Total Score (median [IQR]) | 1.00 [0.00, 3.00] | 1.00 [0.00, 3.00] | 2.00 [1.00, 3.00] |
| Emotional IPV Score (median [IQR]) | 4.00 [4.00, 7.00] | 5.00 [4.00, 7.00] | 5.00 [4.00, 7.00] |
| Physical IPV Score (median [IQR]) | 5.00 [5.00, 7.00] | 5.00 [5.00, 7.00] | 6.00 [5.00, 8.00] |
| ASSIST Tobacco Score (median [IQR]) | 0.00 [0.00, 13.00] | 0.00 [0.00, 14.00] | 0.00 [0.00, 15.00] |
| ASSIST Alcohol Score (median [IQR]) | 0.00 [0.00, 0.00] | 0.00 [0.00, 0.00] | 0.00 [0.00, 0.00] |
| Inflammatory Markers |  |  |  |
| IL-1β pg/mL (median [IQR]) | - | - | 1.05 [0.48, 1.76] |
| IL-6 pg/mL (median [IQR]) | - | - | 1.93 [0.69, 4.08] |
| TNF-α pg/mL (median [IQR]) | - | - | 19.57 [14.48, 28.23] |

**Table S2.** Proportion of missing prenatal exposure data in analysis sample.

| **Exposure** | **# missing** | **% missing** | **n** | **Total N** |
| --- | --- | --- | --- | --- |
| PM_10_ | 20 | 9% | 205 | 225 |
| CO | 43 | 19% | 182 | 225 |
| Benzene | 26 | 12% | 199 | 225 |
| Toluene | 26 | 12% | 199 | 225 |
| NO_2_ | 22 | 10% | 203 | 225 |
| SO_2_ | 22 | 10% | 203 | 225 |
| Maternal smoking (cotinine) | 3 | 1% | 222 | 225 |
| SES assets | 0 | 0% | 225 | 225 |
| food insecurity | 12 | 5% | 213 | 225 |
| SRQ | 13 | 6% | 212 | 225 |
| IPV - emotional | 13 | 6% | 212 | 225 |
| IPV - physical | 13 | 6% | 212 | 225 |
| IPV - sexual | 13 | 6% | 212 | 225 |
| LEQ | 18 | 8% | 207 | 225 |
| EPDS | 14 | 6% | 211 | 225 |
| ASSIST - tobacco | 14 | 6% | 211 | 225 |
| ASSIST - alcohol | 14 | 6% | 211 | 225 |
| Abbreviations: Matter (PM10); Carbon monoxide (CO); Nitrogen dioxide (NO2); Sulfur dioxide (SO2); Socioeconomic Status (SES); Self-Reporting Questionnaire (SRQ-20); Edinburgh Postnatal Depression Scale (EPDS); Life Experiences Questionnaire (LEQ); Intimate Partner Violence (IPV); Alcohol, Smoking, and Substance Involvement Screening Test (ASSIST) | | | | |


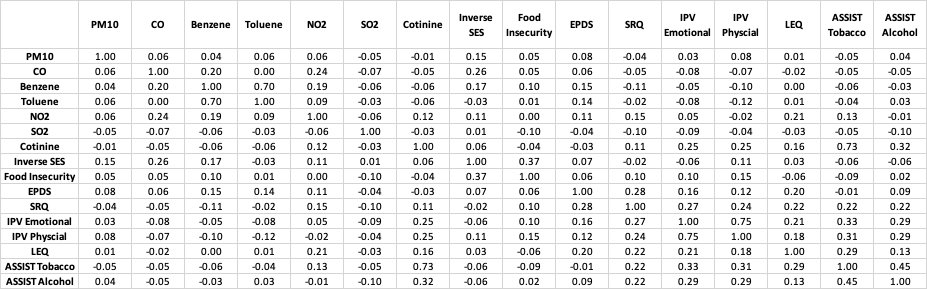


**Table S4.** Pearson correlation matrix with prenatal exposure measurements. Abbreviations: Particulate Matter (PM10); Carbon monoxide (CO); Nitrogen dioxide (NO2); Sulfur dioxide (SO2); Socioeconomic Status (SES); Self-Reporting Questionnaire (SRQ-20); Edinburgh Postnatal Depression Scale (EPDS); Life Experiences Questionnaire (LEQ); Intimate Partner Violence (IPV); Alcohol, Smoking, and Substance Involvement Screening Test (ASSIST).

**Table S4.** Spearman correlation between inflammatory markers at 6 weeks old.

|  | IL-1b | IL-6 | TNF-a |
| --- | --- | --- | --- |
| IL-1b | 1 | 0.39 | 0.05 |
| IL-6 | 0.39 | 1 |  |
| TNF-a | 0.05 | 0.26 | 1 |

**Table S5**. Beta estimates and 95% CIs for individual exposure adjusted linear regression models. The linear regression models were adjusted for maternal HIV status, maternal age, ancestry, and socioeconomic status. Tables shows results from complete case models as well as multiple imputation (MI) models using 5 different random seeds (MI1 to MI5). MI4 models were presented in the main analysis.

|  | Complete Case | MI1 | MI2 | MI3 | MI4 (Analysis Sample) | MI5 |
| --- | --- | --- | --- | --- | --- | --- |
|  | IL-1β | | | | | |
| PM10 Common Confounders | 0 (-0.07, 0.06) | 0.03 (-0.01, 0.07) | 0.03 (-0.01, 0.07) | 0.02 (-0.02, 0.07) | 0.03 (-0.01, 0.07) | 0.04 (-0.01, 0.08) |
| CO Common Confounders | 0.02 (-0.01, 0.04) | 0.01 (-0.01, 0.03) | 0.01 (-0.01, 0.03) | 0.01 (-0.01, 0.03) | 0.01 (-0.01, 0.03) | 0.02 (0, 0.04) |
| Benzene Common Confounders | 0.03 (-0.03, 0.09) | 0.02 (-0.02, 0.06) | 0.02 (-0.02, 0.07) | 0.01 (-0.03, 0.05) | 0.03 (-0.01, 0.07) | 0.02 (-0.03, 0.06) |
| Toluene Common Confounders | 0.01 (-0.04, 0.06) | 0 (-0.03, 0.04) | 0 (-0.03, 0.04) | 0 (-0.03, 0.04) | 0 (-0.03, 0.04) | 0 (-0.04, 0.04) |
| SO2 Common Confounders | 0 (-0.16, 0.17) | -0.02 (-0.12, 0.09) | -0.04 (-0.15, 0.08) | -0.05 (-0.16, 0.06) | -0.04 (-0.15, 0.07) | -0.03 (-0.15, 0.08) |
| NO2 Common Confounders | 0.01 (-0.08, 0.09) | 0 (-0.05, 0.06) | -0.01 (-0.07, 0.05) | 0 (-0.06, 0.06) | 0.01 (-0.05, 0.07) | 0.01 (-0.05, 0.07) |
| Cotinine Common Confounders | 0.01 (-0.04, 0.07) | -0.01 (-0.06, 0.03) | -0.01 (-0.05, 0.04) | -0.01 (-0.05, 0.03) | -0.01 (-0.05, 0.03) | 0 (-0.04, 0.04) |
| ASSIST Tobacco Common Confounders | 0 (-0.06, 0.07) | -0.03 (-0.08, 0.02) | -0.04 (-0.09, 0.01) | -0.03 (-0.08, 0.02) | -0.04 (-0.09, 0.01) | -0.04 (-0.09, 0.01) |
| ASSIST Alcohol Common Confounders | 0 (-0.08, 0.07) | 0 (-0.05, 0.06) | -0.02 (-0.07, 0.04) | 0 (-0.06, 0.06) | -0.01 (-0.06, 0.05) | -0.01 (-0.06, 0.05) |
| EPDS Common Confounders | 0.15 (0.01, 0.29) | 0.11 (0.01, 0.21) | 0.12 (0.02, 0.22) | 0.1 (0.01, 0.2) | 0.1 (0, 0.2) | 0.1 (0, 0.21) |
| SRQ Common Confounders | -0.08 (-0.18, 0.03) | -0.03 (-0.1, 0.05) | -0.05 (-0.12, 0.02) | -0.03 (-0.1, 0.04) | -0.06 (-0.13, 0.01) | -0.04 (-0.11, 0.03) |
| IPV Physical Common Confounders | -0.05 (-0.3, 0.21) | -0.03 (-0.22, 0.15) | -0.05 (-0.24, 0.13) | -0.05 (-0.24, 0.13) | -0.1 (-0.28, 0.08) | -0.04 (-0.23, 0.14) |
| IPV Emotional Common Confounders | -0.06 (-0.31, 0.19) | 0.01 (-0.17, 0.18) | -0.01 (-0.19, 0.16) | -0.04 (-0.21, 0.13) | -0.06 (-0.23, 0.11) | -0.04 (-0.21, 0.13) |
| LEQ Common Confounders | -0.03 (-0.16, 0.11) | -0.05 (-0.15, 0.05) | -0.09 (-0.19, 0.01) | -0.07 (-0.17, 0.03) | -0.08 (-0.18, 0.01) | -0.07 (-0.16, 0.03) |
| SES Common Confounders | 0 (-0.29, 0.29) | -0.03 (-0.23, 0.18) | -0.03 (-0.23, 0.18) | -0.03 (-0.23, 0.18) | -0.03 (-0.23, 0.18) | -0.03 (-0.23, 0.18) |
| Food Insecurity Common Confounders | 0.01 (-0.13, 0.14) | -0.02 (-0.12, 0.08) | -0.01 (-0.11, 0.09) | -0.02 (-0.12, 0.08) | -0.03 (-0.13, 0.07) | 0 (-0.1, 0.1) |
|  | IL-6 | | | | | |
| PM10 Common Confounders | 0.02 (-0.09, 0.14) | 0.05 (-0.01, 0.12) | 0.05 (-0.02, 0.12) | 0.05 (-0.02, 0.12) | 0.03 (-0.04, 0.1) | 0.05 (-0.02, 0.12) |
| CO Common Confounders | 0.02 (-0.03, 0.07) | 0.04 (0.01, 0.07) | 0.02 (-0.01, 0.06) | 0.01 (-0.03, 0.05) | 0.03 (-0.01, 0.07) | 0.03 (-0.01, 0.06) |
| Benzene Common Confounders | 0.06 (-0.05, 0.16) | 0.08 (0.01, 0.15) | 0.06 (-0.02, 0.13) | 0.02 (-0.05, 0.09) | 0.07 (0, 0.14) | 0.06 (-0.01, 0.13) |
| Toluene Common Confounders | 0.05 (-0.04, 0.14) | 0.04 (-0.02, 0.11) | 0.02 (-0.04, 0.09) | 0.02 (-0.05, 0.08) | 0.05 (-0.02, 0.11) | 0.04 (-0.02, 0.11) |
| SO2 Common Confounders | -0.05 (-0.34, 0.23) | -0.14 (-0.32, 0.04) | -0.14 (-0.33, 0.05) | -0.15 (-0.34, 0.04) | -0.15 (-0.34, 0.04) | -0.17 (-0.36, 0.02) |
| NO2 Common Confounders | 0 (-0.15, 0.15) | -0.01 (-0.11, 0.09) | -0.01 (-0.11, 0.09) | -0.01 (-0.11, 0.09) | 0.01 (-0.09, 0.12) | 0 (-0.1, 0.1) |
| Cotinine Common Confounders | 0.02 (-0.08, 0.12) | 0.03 (-0.05, 0.1) | 0.03 (-0.05, 0.1) | 0.03 (-0.04, 0.11) | 0.03 (-0.04, 0.11) | 0.05 (-0.03, 0.12) |
| ASSIST Tobacco Common Confounders | -0.11 (-0.23, 0.01) | -0.05 (-0.14, 0.04) | -0.06 (-0.15, 0.02) | -0.06 (-0.14, 0.03) | -0.06 (-0.14, 0.03) | -0.06 (-0.15, 0.02) |
| ASSIST Alcohol Common Confounders | -0.12 (-0.25, 0.01) | -0.06 (-0.15, 0.04) | -0.08 (-0.17, 0.02) | -0.05 (-0.15, 0.05) | -0.06 (-0.15, 0.04) | -0.07 (-0.17, 0.02) |
| EPDS Common Confounders | 0.08 (-0.17, 0.33) | 0.12 (-0.05, 0.28) | 0.12 (-0.04, 0.29) | 0.11 (-0.06, 0.27) | 0.08 (-0.09, 0.25) | 0.09 (-0.09, 0.27) |
| SRQ Common Confounders | -0.16 (-0.34, 0.02) | -0.08 (-0.2, 0.04) | -0.11 (-0.24, 0.01) | -0.1 (-0.22, 0.02) | -0.13 (-0.25, -0.01) | -0.09 (-0.21, 0.03) |
| IPV Physical Common Confounders | -0.23 (-0.68, 0.21) | -0.12 (-0.44, 0.2) | -0.13 (-0.45, 0.18) | -0.09 (-0.41, 0.22) | -0.2 (-0.51, 0.12) | -0.12 (-0.44, 0.2) |
| IPV Emotional Common Confounders | 0.11 (-0.33, 0.54) | 0.05 (-0.25, 0.35) | 0 (-0.29, 0.3) | -0.01 (-0.31, 0.29) | -0.04 (-0.34, 0.26) | -0.02 (-0.32, 0.28) |
| LEQ Common Confounders | -0.2 (-0.44, 0.04) | -0.11 (-0.27, 0.06) | -0.21 (-0.38, -0.04) | -0.15 (-0.32, 0.01) | -0.16 (-0.33, 0) | -0.16 (-0.32, 0.01) |
| SES Common Confounders | -0.23 (-0.74, 0.27) | -0.01 (-0.36, 0.34) | -0.01 (-0.36, 0.34) | -0.01 (-0.36, 0.34) | -0.01 (-0.36, 0.34) | -0.01 (-0.36, 0.34) |
| Food Insecurity Common Confounders | 0 (-0.24, 0.23) | -0.05 (-0.22, 0.11) | -0.05 (-0.22, 0.12) | -0.07 (-0.24, 0.1) | -0.07 (-0.24, 0.1) | -0.04 (-0.21, 0.13) |
|  | TNF-α | | | | | |
| PM10 Common Confounders | -0.08 (-0.16, 0) | -0.04 (-0.1, 0.01) | -0.03 (-0.08, 0.03) | -0.03 (-0.08, 0.03) | -0.03 (-0.09, 0.02) | -0.04 (-0.09, 0.01) |
| CO Common Confounders | 0.02 (-0.02, 0.05) | 0.01 (-0.01, 0.04) | 0.02 (-0.01, 0.04) | 0.01 (-0.02, 0.04) | 0.02 (-0.01, 0.05) | 0.01 (-0.01, 0.04) |
| Benzene Common Confounders | 0.05 (-0.03, 0.12) | 0.05 (-0.01, 0.11) | 0.05 (-0.01, 0.1) | 0.05 (-0.01, 0.1) | 0.06 (0, 0.12) | 0.04 (-0.01, 0.1) |
| Toluene Common Confounders | 0.03 (-0.03, 0.1) | 0.02 (-0.03, 0.07) | 0.02 (-0.03, 0.07) | 0.02 (-0.02, 0.07) | 0.04 (-0.01, 0.09) | 0.02 (-0.03, 0.07) |
| SO2 Common Confounders | -0.13 (-0.34, 0.08) | -0.1 (-0.24, 0.04) | -0.09 (-0.23, 0.06) | -0.1 (-0.25, 0.04) | -0.11 (-0.25, 0.04) | -0.1 (-0.25, 0.04) |
| NO2 Common Confounders | 0.06 (-0.05, 0.17) | -0.03 (-0.11, 0.05) | -0.05 (-0.12, 0.03) | -0.02 (-0.1, 0.06) | -0.04 (-0.11, 0.04) | -0.04 (-0.12, 0.04) |
| Cotinine Common Confounders | 0.02 (-0.06, 0.1) | -0.02 (-0.08, 0.03) | -0.02 (-0.07, 0.04) | -0.02 (-0.08, 0.04) | -0.02 (-0.08, 0.04) | -0.01 (-0.07, 0.05) |
| ASSIST Tobacco Common Confounders | 0.04 (-0.05, 0.13) | 0 (-0.07, 0.06) | 0 (-0.07, 0.06) | -0.01 (-0.07, 0.06) | 0 (-0.06, 0.07) | -0.01 (-0.08, 0.05) |
| ASSIST Alcohol Common Confounders | 0.05 (-0.05, 0.15) | -0.01 (-0.08, 0.07) | 0 (-0.07, 0.08) | 0.01 (-0.06, 0.08) | 0.01 (-0.06, 0.08) | -0.03 (-0.1, 0.04) |
| EPDS Common Confounders | 0.16 (-0.02, 0.35) | 0.08 (-0.04, 0.21) | 0.12 (0, 0.25) | 0.13 (0.01, 0.26) | 0.09 (-0.04, 0.22) | 0.09 (-0.04, 0.23) |
| SRQ Common Confounders | -0.07 (-0.2, 0.06) | -0.13 (-0.22, -0.04) | -0.14 (-0.23, -0.04) | -0.14 (-0.23, -0.04) | -0.16 (-0.25, -0.07) | -0.14 (-0.24, -0.05) |
| IPV Physical Common Confounders | 0.11 (-0.22, 0.45) | -0.05 (-0.29, 0.19) | -0.05 (-0.29, 0.19) | -0.03 (-0.27, 0.21) | -0.06 (-0.29, 0.18) | -0.06 (-0.3, 0.18) |
| IPV Emotional Common Confounders | 0.17 (-0.16, 0.49) | 0.02 (-0.21, 0.25) | 0 (-0.22, 0.23) | 0.02 (-0.21, 0.25) | 0 (-0.22, 0.23) | -0.01 (-0.24, 0.21) |
| LEQ Common Confounders | 0.12 (-0.06, 0.3) | 0.02 (-0.11, 0.15) | -0.02 (-0.14, 0.11) | -0.01 (-0.14, 0.12) | 0.02 (-0.1, 0.15) | 0.03 (-0.1, 0.16) |
| SES Common Confounders | -0.12 (-0.5, 0.25) | 0.02 (-0.24, 0.29) | 0.02 (-0.24, 0.29) | 0.02 (-0.24, 0.29) | 0.02 (-0.24, 0.29) | 0.02 (-0.24, 0.29) |
| Food Insecurity Common Confounders | -0.05 (-0.22, 0.13) | -0.04 (-0.17, 0.09) | -0.03 (-0.15, 0.1) | -0.03 (-0.15, 0.1) | -0.05 (-0.18, 0.07) | -0.04 (-0.17, 0.09) |
| Abbreviations: Matter (PM10); Carbon monoxide (CO); Nitrogen dioxide (NO2); Sulfur dioxide (SO2); Socioeconomic Status (SES); Self-Reporting Questionnaire (SRQ-20); Edinburgh Postnatal Depression Scale (EPDS); Life Experiences Questionnaire (LEQ); Intimate Partner Violence (IPV); Alcohol, Smoking, and Substance Involvement Screening Test (ASSIST) | | | | | | |

**Table S6.** Beta estimates and 95% CIs for individual exposure adjusted linear regression models. The common confounder linear regression models were adjusted for maternal HIV status, maternal age, ancestry, and socioeconomic status. Extended confounder set models using indoor air pollutant exposures were additionally adjusted principal components of psychosocial factors, and vice versa.

|  | IL-1β | IL-6 | TNF-α |
| --- | --- | --- | --- |
| PM10 Common Confounders | 0.03 (-0.01, 0.07) | 0.03 (-0.04, 0.1) | -0.03 (-0.09, 0.02) |
| PM10 Extended Confounder Set | 0.03 (-0.01, 0.07) | 0.03 (-0.04, 0.11) | -0.03 (-0.09, 0.02) |
| CO Common Confounders | 0.01 (-0.01, 0.03) | 0.03 (-0.01, 0.07) | 0.02 (-0.01, 0.05) |
| CO Extended Confounder Set | 0.01 (-0.01, 0.03) | 0.03 (-0.01, 0.06) | 0.02 (-0.01, 0.05) |
| Benzene Common Confounders | 0.03 (-0.01, 0.07) | 0.07 (0, 0.14) | 0.06 (0, 0.12) |
| Benzene Extended Confounder Set | 0.03 (-0.02, 0.07) | 0.07 (0, 0.14) | 0.06 (0.01, 0.12) |
| Toluene Common Confounders | 0 (-0.03, 0.04) | 0.05 (-0.02, 0.11) | 0.04 (-0.01, 0.09) |
| Toluene Extended Confounder Set | 0 (-0.04, 0.04) | 0.05 (-0.02, 0.12) | 0.04 (-0.01, 0.09) |
| SO2 Common Confounders | -0.04 (-0.15, 0.07) | -0.15 (-0.34, 0.04) | -0.11 (-0.25, 0.04) |
| SO2 Extended Confounder Set | -0.05 (-0.16, 0.07) | -0.17 (-0.36, 0.02) | -0.13 (-0.27, 0.02) |
| NO2 Common Confounders | 0.01 (-0.05, 0.07) | 0.01 (-0.09, 0.12) | -0.04 (-0.11, 0.04) |
| NO2 Extended Confounder Set | 0.01 (-0.05, 0.07) | 0.03 (-0.08, 0.13) | -0.03 (-0.12, 0.05) |
| Cotinine Common Confounders | -0.01 (-0.05, 0.03) | 0.03 (-0.04, 0.11) | -0.02 (-0.08, 0.04) |
| Cotinine Extended Confounder Set | -0.01 (-0.05, 0.04) | 0.04 (-0.03, 0.12) | -0.02 (-0.07, 0.04) |
| ASSIST Tobacco Common Confounders | -0.04 (-0.09, 0.01) | -0.06 (-0.14, 0.03) | 0 (-0.06, 0.07) |
| ASSIST Tobacco Extended Confounder Set | -0.03 (-0.08, 0.02) | -0.05 (-0.14, 0.03) | 0 (-0.07, 0.06) |
| ASSIST Alcohol Common Confounders | -0.01 (-0.06, 0.05) | -0.06 (-0.15, 0.04) | 0.01 (-0.06, 0.08) |
| ASSIST Alcohol Extended Confounder Set | -0.01 (-0.06, 0.05) | -0.06 (-0.16, 0.04) | 0.01 (-0.07, 0.08) |
| EPDS Common Confounders | 0.1 (0, 0.2) | 0.08 (-0.09, 0.25) | 0.09 (-0.04, 0.22) |
| EPDS Extended Confounder Set | 0.08 (-0.02, 0.18) | 0.04 (-0.13, 0.21) | 0.07 (-0.06, 0.2) |
| SRQ Common Confounders | -0.06 (-0.13, 0.01) | -0.13 (-0.25, -0.01) | -0.16 (-0.25, -0.07) |
| SRQ Extended Confounder Set | -0.06 (-0.13, 0.02) | -0.13 (-0.26, -0.01) | -0.16 (-0.25, -0.07) |
| IPV Physical Common Confounders | -0.1 (-0.28, 0.08) | -0.2 (-0.51, 0.12) | -0.06 (-0.29, 0.18) |
| IPV Physical Extended Confounder Set | -0.1 (-0.28, 0.09) | -0.17 (-0.49, 0.14) | -0.02 (-0.26, 0.22) |
| IPV Emotional Common Confounders | -0.06 (-0.23, 0.11) | -0.04 (-0.34, 0.26) | 0 (-0.22, 0.23) |
| IPV Emotional Extended Confounder Set | -0.06 (-0.23, 0.12) | -0.03 (-0.33, 0.26) | 0.01 (-0.22, 0.24) |
| LEQ Common Confounders | -0.08 (-0.18, 0.01) | -0.16 (-0.33, 0) | 0.02 (-0.1, 0.15) |
| LEQ Extended Confounder Set | -0.09 (-0.18, 0.01) | -0.18 (-0.34, -0.01) | 0.02 (-0.1, 0.15) |
| SES Common Confounders | -0.03 (-0.23, 0.18) | -0.01 (-0.36, 0.34) | 0.02 (-0.24, 0.29) |
| SES Extended Confounder Set | -0.08 (-0.29, 0.13) | -0.1 (-0.47, 0.27) | 0.03 (-0.25, 0.31) |
| Food Insecurity Common Confounders | -0.03 (-0.13, 0.07) | -0.07 (-0.24, 0.1) | -0.05 (-0.18, 0.07) |
| Food Insecurity Extended Confounder Set | -0.03 (-0.13, 0.07) | -0.09 (-0.26, 0.08) | -0.07 (-0.2, 0.06) |
| Abbreviations: Matter (PM10); Carbon monoxide (CO); Nitrogen dioxide (NO2); Sulfur dioxide (SO2); Socioeconomic Status (SES); Self-Reporting Questionnaire (SRQ-20); Edinburgh Postnatal Depression Scale (EPDS); Life Experiences Questionnaire (LEQ); Intimate Partner Violence (IPV); Alcohol, Smoking, and Substance Involvement Screening Test (ASSIST) | | | |

**Table S7.** Descriptive statistics (Median (IQR)) of indoor air pollutant and psychosocial factor exposures, demographic characteristics and inflammatory markers in Self-Organizing Map (SOM) exposure clusters.

|  | **SOM Cluster** | | | |
| --- | --- | --- | --- | --- |
|  | **1** | **2** | **3** | **4** |
| N (%) | 88 (40.11) | 49 (21.78) | 41 (18.22) | 47 (20.89) |
| Maternal Age (mean (SD)) | 27.49 (5.80) | 29.66 (5.05) | 24.06 (4.94) | 28.98 (6.73) |
| Male Child (%) | 52 (59.1) | 28 (57.1) | 29 (70.7) | 21 (44.7) |
| Mixed Ancestry (%) | 20 (22.7) | 8 (16.3) | 36 (87.8) | 26 (55.3) |
| Mother HIV Positive (%) | 39 (44.3) | 32 (65.3) | 7 (17.1) | 17 (36.2) |
| Indoor Air Pollutants |  |  |  |  |
| PM10 µg/m3 (median [IQR]) | 31.99 [15.09, 69.16] | 44.25 [17.03, 71.68] | 48.04 [13.94, 66.42] | 44.05 [15.32, 70.71] |
| CO mg/m3 (median [IQR]) | 0.00 [0.00, 0.00] | 120.00 [0.00, 1060.00] | 0.00 [0.00, 120.00] | 0.00 [0.00, 0.00] |
| Benzene µg/m3 (median [IQR]) | 3.15 [1.27, 4.68] | 47.01 [22.78, 100.57] | 5.48 [2.77, 14.27] | 2.73 [1.12, 5.49] |
| Toluene µg/m3 (median [IQR]) | 11.12 [4.90, 18.14] | 53.72 [27.01, 403.17] | 20.78 [10.63, 63.24] | 9.42 [3.79, 20.94] |
| NO2 µg/m3 (median [IQR]) | 4.41 [1.54, 7.97] | 10.57 [5.36, 19.82] | 9.24 [3.97, 12.61] | 5.70 [3.64, 9.90] |
| SO2 µg/m3 (median [IQR]) | 0.00 [0.00, 0.12] | 0.00 [0.00, 0.00] | 0.00 [0.00, 0.25] | 0.00 [0.00, 0.08] |
| Psychosocial Factors |  |  |  |  |
| Urine Cotinine ng/ml (median [IQR]) | 19.00 [10.00, 63.02] | 30.00 [13.70, 61.00] | 500.00 [500.00, 500.00] | 500.00 [43.20, 500.00] |
| SES Asset Sum (median [IQR]) | 7.00 [5.75, 8.00] | 5.00 [4.00, 7.00] | 7.00 [6.00, 8.00] | 7.00 [5.00, 8.00] |
| Food Insecurity Total Score (median [IQR]) | 0.00 [0.00, 1.00] | 2.00 [0.00, 4.00] | 0.00 [0.00, 0.00] | 1.00 [0.00, 4.00] |
| SRQ-20 Total Score (median [IQR]) | 4.00 [1.00, 7.00] | 2.00 [1.00, 5.00] | 5.00 [3.00, 8.00] | 8.00 [5.00, 12.00] |
| Emotional IPV Score (median [IQR]) | 4.00 [4.00, 5.00] | 4.00 [4.00, 6.00] | 5.00 [4.00, 7.00] | 11.00 [8.50, 13.50] |
| Physical IPV Score (median [IQR]) | 5.00 [5.00, 6.00] | 6.00 [5.00, 6.00] | 6.00 [5.00, 7.00] | 12.00 [9.00, 15.00] |
| LEQ Total Score (median [IQR]) | 1.00 [0.00, 2.25] | 1.00 [0.00, 2.00] | 2.00 [1.00, 4.00] | 3.00 [1.00, 4.50] |
| EPDS Total Score (median [IQR]) | 9.00 [6.00, 12.00] | 12.00 [9.00, 14.00] | 10.00 [6.00, 16.00] | 10.00 [8.00, 18.00] |
| ASSIST Tobacco Score (median [IQR]) | 0.00 [0.00, 0.00] | 0.00 [0.00, 0.00] | 21.00 [18.00, 24.00] | 13.00 [0.00, 24.00] |
| ASSIST Alcohol Score (median [IQR]) | 0.00 [0.00, 0.00] | 0.00 [0.00, 0.00] | 3.00 [0.00, 12.00] | 0.00 [0.00, 15.00] |
| Inflammatory Markers |  |  |  |  |
| IL1b (median [IQR]) | 1.00 [0.42, 1.79] | 1.08 [0.72, 1.78] | 1.00 [0.45, 1.43] | 1.11 [0.49, 1.68] |
| IL6 (median [IQR]) | 1.70 [0.58, 3.80] | 3.10 [1.22, 7.12] | 1.69 [0.68, 3.96] | 1.69 [0.49, 3.16] |
| TNFa (median [IQR]) | 19.23 [14.61, 26.61] | 23.38 [16.61, 32.08] | 20.68 [13.43, 29.74] | 17.55 [13.37, 22.62] |

**Table S8.** Results from linear regression models using SOM cluster as the exposure, adjusted for maternal age, maternal HIV status, and ancestry.

| IL1b | |
| --- | --- |
| SOM Cluster | Beta (95% CI) |
| 1 (Reference) | - |
| 2 | 0.13 (-0.03, 0.29) |
| 3 | -0.02 (-0.22, 0.18) |
| 4 | 0.02 (-0.15, 0.19) |
| IL6 | |
| SOM Cluster | Beta (95% CI) |
| 1 (Reference) | - |
| 2 | **0.35 (0.07, 0.62)** |
| 3 | 0.15 (-0.18, 0.48) |
| 4 | -0.06 (-0.35, 0.23) |
| TNFa | |
| SOM Cluster | Beta (95% CI) |
| 1 (Reference) | - |
| 2 | **0.22 (0.01, 0.43)** |
| 3 | 0.10 (-0.15, 0.36) |
| 4 | -0.01 (-0.23. 0.21) |

**Figures**


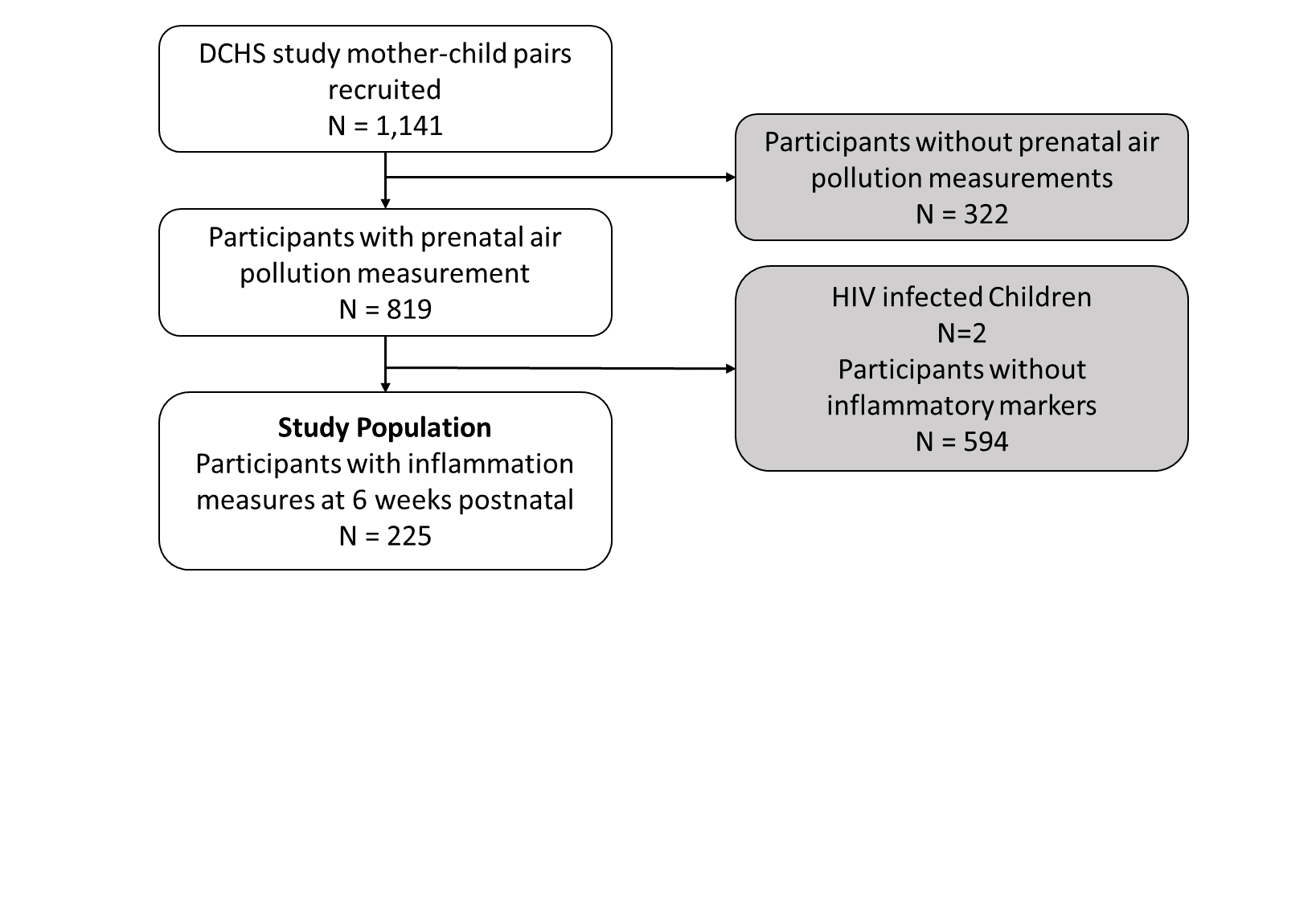


**Figure S1.** Study population flow diagram.


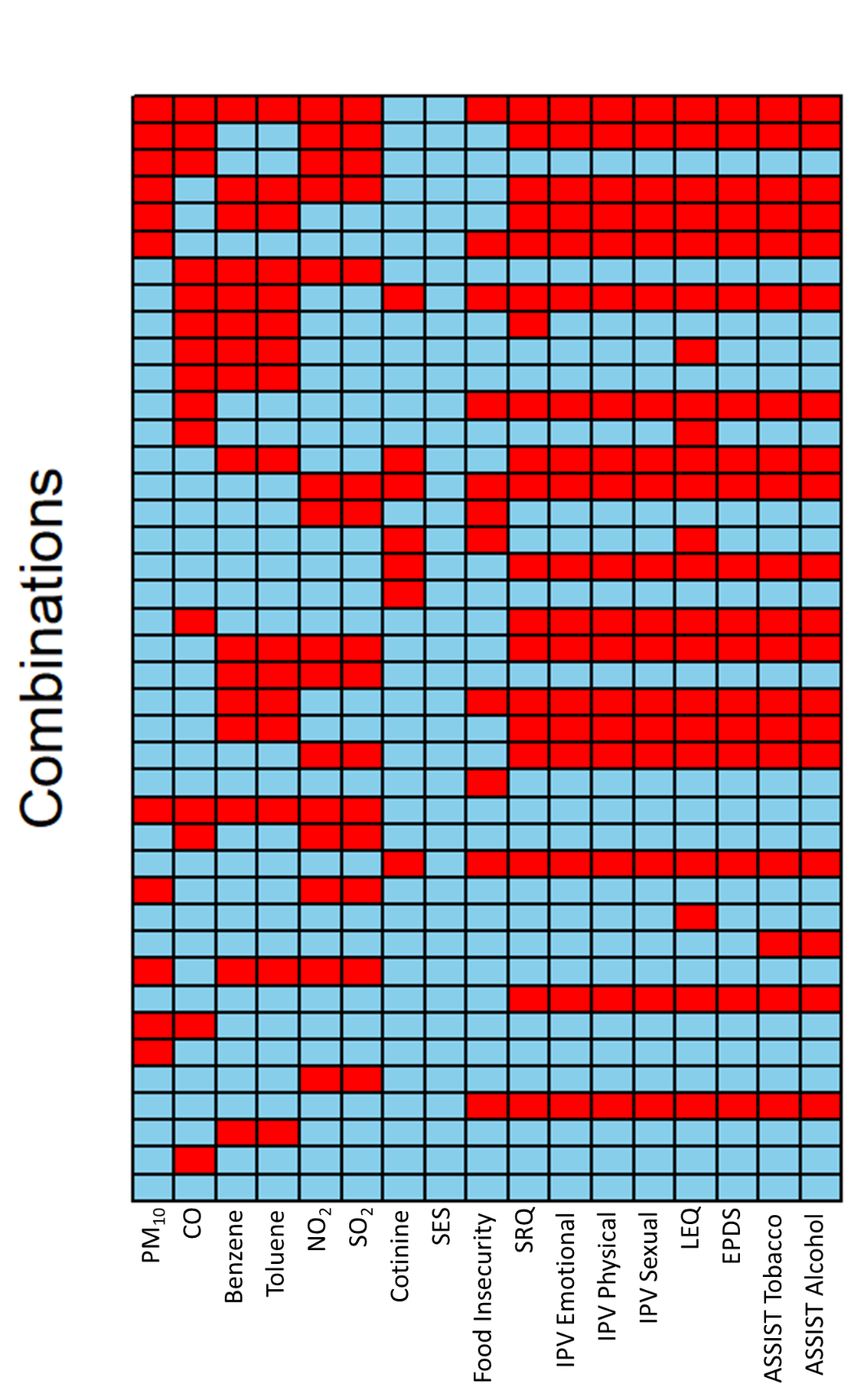


**Figure S2.** Combinations of missingness patterns of exposure variables. Each row is a missingness pattern where red indicates that variable is missing, and blue indicates not missing.


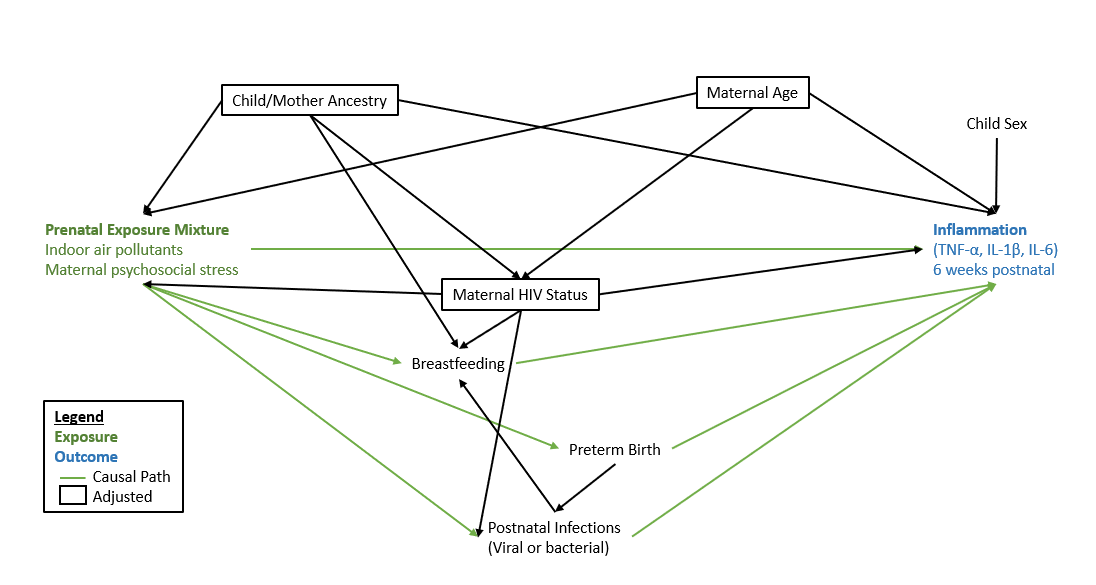


**Figure S3.** Directed Acyclic Graph (DAG) of underlying causal pathways between prenatal exposure to indoor air pollutants and psychosocial factors including socioeconomic status, and inflammatory markers at 6 weeks old.

**A.**


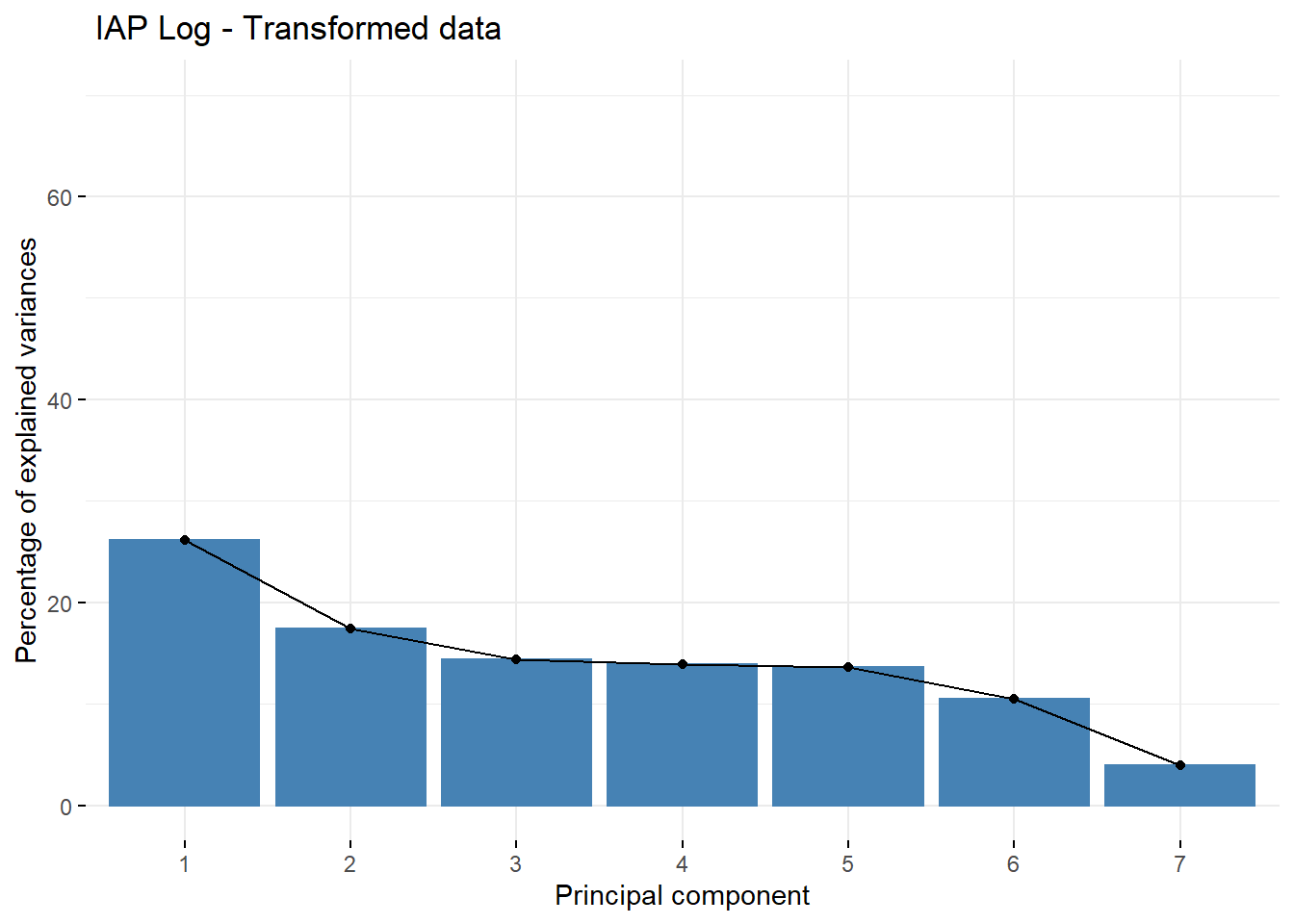


**B.**


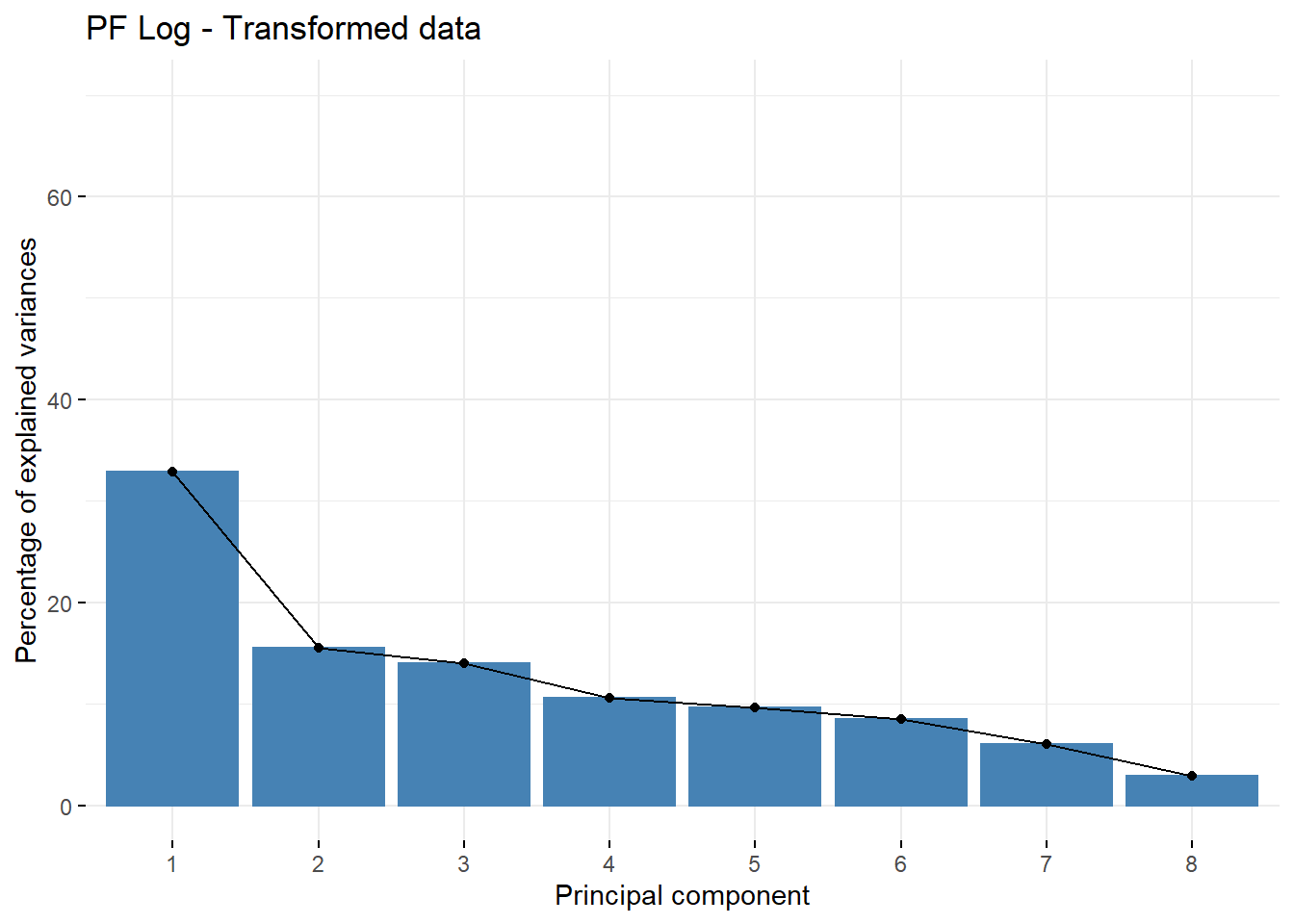


**Figure S4.** Scree plots detailing the percentage of explained variance for each principal component. **A.** Principal components of indoor air pollution variables. **B.** Principal components of psychosocial factor variables.

**A.** SRQ/IL-6 **B.** LEQ/IL-6 **C.** Benzene/TNF-α  **D.** SRQ/TNF-α


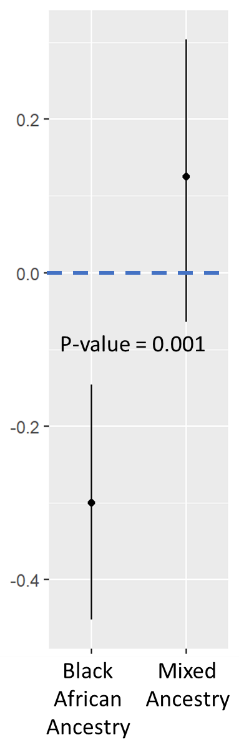

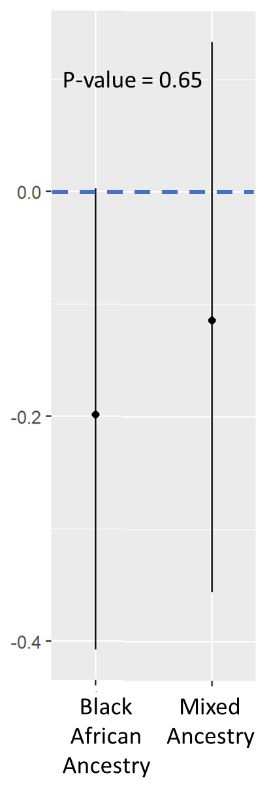

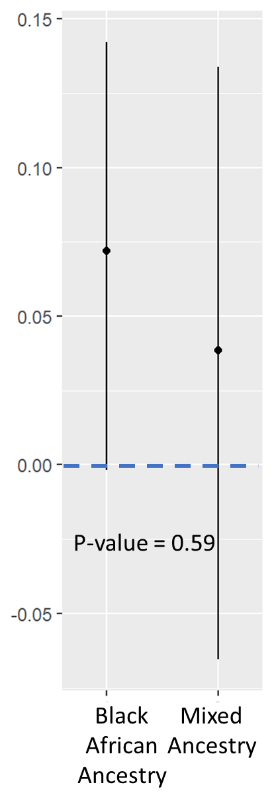

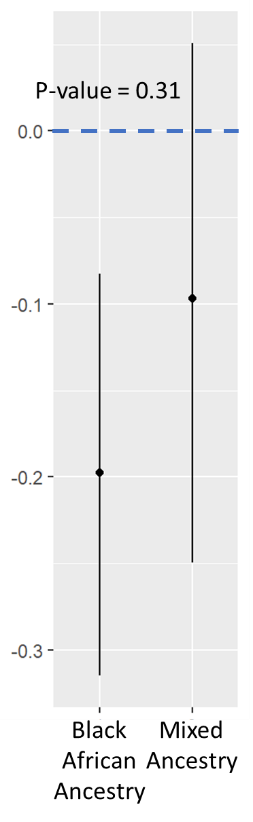


**Figure S5**. Interaction of selected exposures and participant ancestry. Effect estimates presented of select exposures among ancestry groups. All models adjusted for maternal age, maternal HIV, and socioeconomic status.

**A.** SRQ/IL-6 **B.** LEQ/IL-6 **C.** Benzene/TNF-α **D.** SRQ/TNF-α


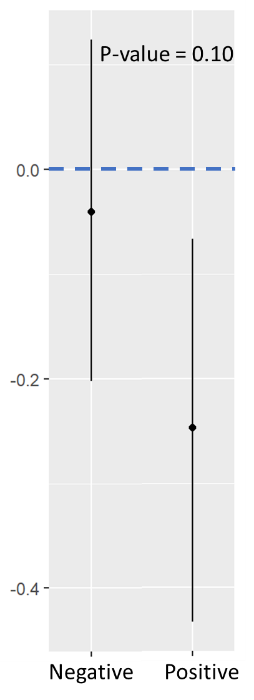

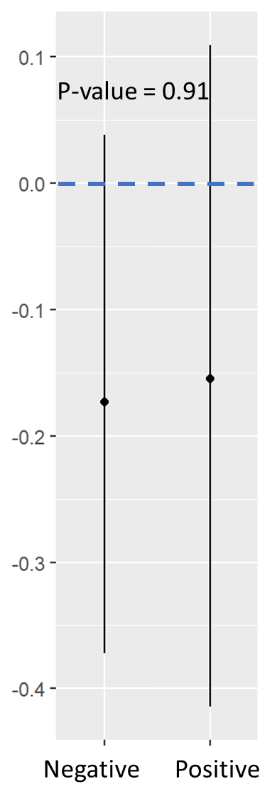

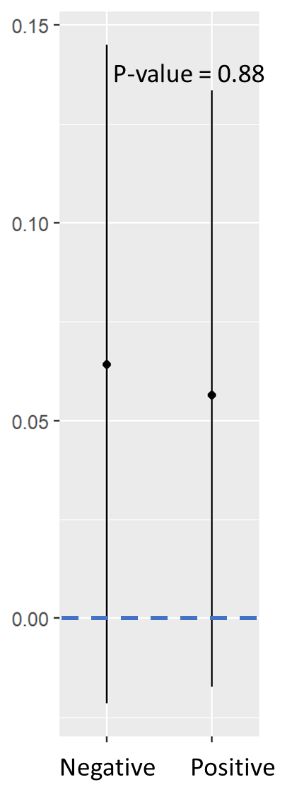

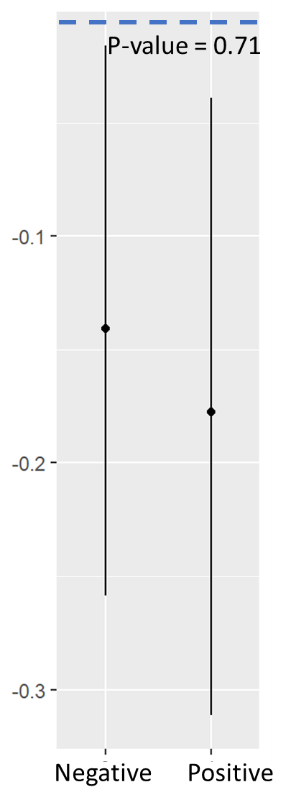


**Figure S6**. Interaction of selected exposures and participant HIV status. Effect estimates presented of select exposures among HIV status groups. All models adjusted for maternal age, ancestry, and socioeconomic status.
